# Gas6 expression is reduced in advanced breast cancers

**DOI:** 10.1038/s41698-020-0116-z

**Published:** 2020-04-24

**Authors:** Ayman M. Ibrahim, Zane Gray, Angelica M. Gomes, Leann Myers, Fariba Behbod, Heather L. Machado

**Affiliations:** 1 Department of Biochemistry and Molecular Biology, Tulane Cancer Center, Tulane School of Medicine, New Orleans, LA USA; 2 Department of Zoology, Faculty of Science, Cairo University, Giza, Egypt; 3 Department of Biostatistics and Data Science, Tulane University School of Public Health and Tropical Medicine, New Orleans, LA USA; 4 Department of Pathology and Laboratory Medicine, University of Kansas Medical Center, Kansas City, KS USA

**Keywords:** Breast cancer, Targeted therapies

## Abstract

Growth arrest-specific gene 6 (Gas6) is a cytokine that binds to receptor tyrosine kinases Tyro3, Axl, and Mer. Numerous studies have suggested that macrophage-derived Gas6 interacts with Axl to promote cancer progression, and Axl has been associated with poor clinical outcome. However, the expression and relevance of Gas6 in human breast cancer patients has not been studied. Analysis of tissue microarrays showed that Gas6 was highly expressed in ductal carcinoma in situ (DCIS) but markedly decreased in invasive breast cancer. Gas6 and Axl were weakly correlated, suggesting that their functions may not exclusively rely on each other. Analyses of publicly available databases showed significantly improved overall and relapse-free survival in patients with high Gas6 mRNA, particularly in luminal A breast cancers. These findings indicate that tumor-derived Gas6 is not overexpressed in invasive breast cancer, and may not be a negative prognostic factor in human breast cancer.

## Introduction

Gas6 is a vitamin K-dependent cytokine that binds to a family of receptor tyrosine kinases that includes Tyro3, Axl, and Mer (TAMR family), with 100–1000 times higher affinity for Axl^1,2^. Gas6/TAMR signaling is a critical component of the innate immune response, functions in phagocytic clearance of apoptotic cells and is an important thrombosis factor. More recently, Gas6 was shown to modulate different cellular events such as proliferation, survival and invasion in vitro^3–5^. In vitro studies have suggested that Gas6/Axl signaling promotes tumor cell survival and invasion, such as osteosarcoma^6^, hepatocellular carcinoma^7^, renal cell carcinoma^8^, and lung cancer^9^. In breast cancer, Axl is highly expressed in triple negative breast cancer (TNBC) cell lines^10^ and is correlated with poor clinical outcome (reviewed in ref. ^11^). In addition, studies using mouse models have shown that macrophage-derived Gas6 may have a tumor-promoting role in cancer progression^12^. In contrast, other studies revealed Gas6-independent functions of Axl including regulation of epithelial–mesenchymal–transition and breast cancer metastasis^10,13,14^. Axl can be activated by other RTKs, including EGFR and VEGFR2, and has been associated with resistance to targeted therapies in lung, pancreatic, and breast cancers^15–17^.

In the past decade, there have been tremendous efforts in targeting Axl, and more recently Gas6, as a therapeutic strategy for metastatic breast cancer. However, few studies have addressed the expression of Gas6 and relation to overall survival in human breast cancers. In this study, we assessed the expression pattern of Gas6 at different stages and subtypes of human breast cancer. To our surprise, tumor-derived Gas6 significantly decreased in breast cancer as compared with normal tissues, and overall survival and relapse-free survival (RFS) was significantly improved in breast cancers with high Gas6 expression. In support, Gas6 mRNA and protein expression was reduced in luminal B, Her2^+^, and TNBC, which may suggest that Gas6 overexpression is not a negative prognostic factor.

## Results

### Gas6 expression declines in invasive breast tumors

**Fig. 1:**
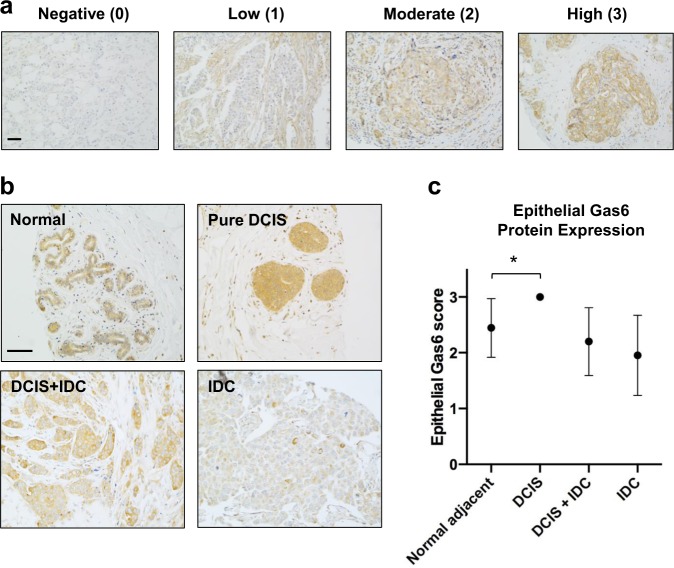
Gas6 expression in normal breast and invasive breast cancer. **a** Representative images of epithelial Gas6 staining and the corresponding score assigned as negative (0), low (1), moderate (2), or high (3). **b** Immunohistochemical analysis of Gas6 in normal breast tissue and different stages of cancer, showing the localization and the differential expression of Gas6. **c** Graph depicts epithelial Gas6 score in the epithelium of normal breast and different stages of breast cancer (normal: *n* = 9, pure DCIS: *n* = 12, DCIS/IDC: *n* = 44, IDC: *n* = 144). Error bars are SEM, *p = 0.008 (Chi-squared test). Scale bars = 100 μm.

We previously reported that Gas6 was expressed in macrophages and pre-invasive epithelial cells of ductal carcinoma in situ (DCIS)^18^, the non-obligatory precursor of invasive ductal carcinoma (IDC). To expand these studies, we stained two tissue microarrays (TMAs) (Table 1) with an antibody that recognizes human Gas6, and scored epithelial staining based on intensity and the number of stained cells. Antibody specificity was validated by staining NCI-H226 cells (positive control) or HT29 cells (negative control) (Supplementary Fig. 1a). For TMAs, epithelial Gas6 expression was scored on a scale from 0 to 3 and represented as negative (0), low (1), moderate (2), or high (3) (Fig. 1a). In support of our previous studies in the mouse mammary gland^19^, Gas6 was highly expressed in the ductal epithelium of normal breast (adjacent normal). Compared to normal tissue, there was a significant increase in Gas6 protein in pure DCIS. On the other hand, Gas6 expression was decreased in DCIS with adjacent IDC (DCIS + IDC), with the lowest expression in IDC alone (Fig. 1b, c, and Supplementary Fig. 1b). Notably, Gas6 staining was observed throughout the cytoplasm and concentrated near the ductal lumens in normal breast, whereas Gas6 appeared to be localized to specific cytoplasmic compartments in IDC. Stromal Gas6 expression was unchanged amongst groups (Supplementary Fig. 1c). We also analyzed Gas6 mRNA in the GEPIA breast cancer dataset, an online platform that employs data from the TCGA and GTex projects^20^. Interestingly, multiple adjacent normal female reproductive tissues including breast, uterus, ovary, and cervix expressed higher levels of Gas6 mRNA than their tumor counterparts (Supplementary Fig. 2a). Further analysis revealed a statistically significant reduction in Gas6 (RNA transcripts per million) in breast cancer (*n* = 1085) compared with normal (*n* = 291) (Supplementary Fig. 2b). These data suggest that Gas6 mRNA is highly expressed in normal female reproductive tissues but decreased in invasive cancers.

**Table 1:** Histopathological characteristics of patient cohort used in the IHC analysis.

Parameter	Pure DCIS	DCIS + IDC	IDC
	(*n* = 12) (%)	(*n* = 44) (%)	(*n* = 100) (%)
Age (yrs)			
Range	36–81	41–91	29–95
Mean	53 (13.3)	58.16 (12.11)	50.65 (12.3)
Tumor grade			
Grade I	8.3%	0%	6%
Grade II	66.7%	45.7%	72.7%
Grade III	25%	54.3%	21.3%
ER status			
Positive	9%	73.8%	68%
Negative	91%	26.2%	32%
PR status			
Positive	18.2%	61.7%	57%
Negative	81.8%	39.3%	43%
Her2 status			
Positive	36.4%	23.23%	40%
Negative	63.6%	76.76%	60%
Ki67			
Low	63.5%	62.4%	64%
High	36.5%	38.6%	36%

The cohort included patients with DCIS, IDC, and with different molecular subtypes.

### Gas6 expression is decreased in luminal B, Her2^+^, and basal-like breast cancers

**Fig. 2:**
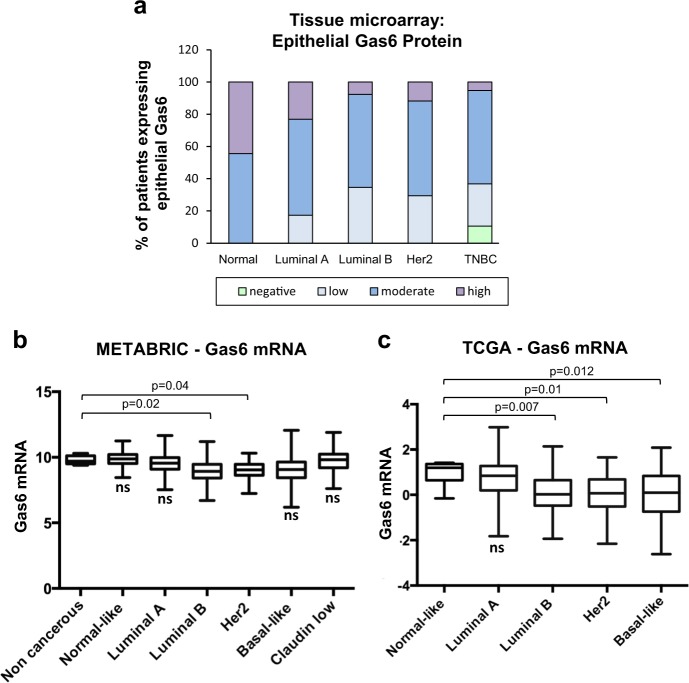
Gas6 expression across breast cancer subtypes. **a** Graph depicts quantitation Gas6 protein staining of TMAs, expressed as the percentage (%) of patients expressing epithelial Gas6 protein in different molecular subtypes: normal breast (*n* = 9), luminal A (*n* = 39), luminal B (*n* = 18), Her2^+^ (*n* = 19), and TNBC (*n* = 19). **b** Mean Gas6 mRNA expression across distinct molecular subtypes relative to non-cancer and normal-like breast tissues, using METABRIC dataset (non-cancerous, *n* = 6; normal-like cancers, *n* = 140; luminal A, *n* = 679; luminal B, *n* = 461; Her2, *n* = 219; basal-like, *n* = 198; claudin low, *n* = 199). **c** Mean Gas6 mRNA expression across distinct molecular subtypes relative to normal-like breast tissues, using TCGA data set (normal-like cancers, *n* = 8; luminal A, *n* = 212; luminal B, *n* = 119; Her2, *n* = 55; basal-like, *n* = 87). Middle line is median, box borders are 25th to 75th percentiles and bars are minimum and maximum values. Significant *p* values are depicted in graphs **b**–**c** from one-way ANOVA followed by post hoc Dunnett’s multiple comparison test. ns not significant.

To determine whether Gas6 is preferentially expressed in a particular breast cancer subtype, we categorized patient samples from the TMAs as luminal A, luminal B, Her2^+^ and TNBC, based on clinical attributes including hormone receptor status, Her2 status, and Ki67 expression. The majority of patient samples expressed moderate levels of epithelial Gas6 regardless of subtype, although a subset of patients with TNBC did not express Gas6 (Fig. 2a). Interestingly, luminal A breast cancers, which generally have a favorable outcome, showed the largest percentage of patients with high Gas6 expression (Fig. 2a and Supplementary Fig. 3a). The localization and pattern of Gas6 staining was consistent among all breast cancer subtypes. Stromal protein expression of Gas6 was also quantified, and showed no distinct difference between subtypes, nonetheless, a small subset of luminal B and Her2^+^ patients had the highest Gas6 expression (Supplementary Fig. 3b)

Next, we used cBioportal^21^ to analyze breast cancer patients from the METABRIC (*n* = 1904)^22^ and TCGA (*n* = 481)^23^ data sets. Patients in each data set were grouped by PAM50 score, a tumor profiling test of 50 known genes in breast cancer, to plot Gas6 mRNA levels across distinct molecular subtypes. In METABRIC data, patients with luminal B or Her2^+^ expressed significantly lower levels of Gas6 mRNA relative to non-cancerous breast tissue (Fig. 2b). Similarly, analysis of TCGA data showed that patients with luminal B, Her2^+^, and basal-like (which includes TNBC) tumors expressed significantly lower levels of Gas6 mRNA, relative to normal-like cancers (Fig. 2c). These results suggest that Gas6 is expressed amongst all breast cancer subtypes but is lowest in breast cancers that are commonly characterized by poor outcome.

### Gas6 expression weakly correlates with Axl in invasive breast cancer

**Fig. 3:**
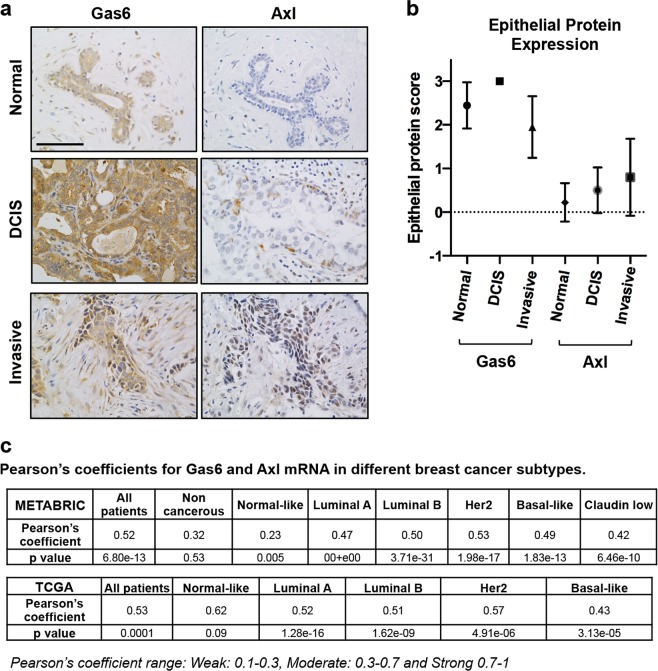
Differential expression of Gas6 and Axl. **a** Images depict immunohistochemical staining using antibodies to Gas6 or Axl in normal (*n* = 9), DCIS (*n* = 12) and invasive ductal carcinoma (*n* = 100) from the same patients. Scale bar = 100 μm. **b** Graph shows quantitation of Gas6 or Axl staining represented by score (0–3) in normal, DCIS and invasive breast tissues. Error bars are SEM. **c** Tables represent Pearson’s coefficients, and the corresponding *p* value, between Gas6 mRNA, and Axl mRNA in METABRIC and TCGA data sets, using either all patients data or PAM50-classfied subtypes.

Numerous studies in breast cancer models have shown tumor-promoting functions of Gas6 through interactions of the Axl receptor^4,6,24^. In contrast, a recent study using a mouse model of Her2^+^ breast cancer (MMTV-Neu) showed that Axl-mediated metastasis was ligand independent^25^. Therefore, we next asked whether there was an association between Gas6 and Axl protein expression in clinical samples. To address this question, consecutive sections from the same patients were stained with antibodies to either Gas6 or Axl, as shown in Fig. 3a. Antibody specificity was validated by staining H1299 cells (positive control) or Jurkat cells (negative control) (Supplementary Fig. 4). As with Gas6 scoring (Fig. 1a), Axl expression was scored based on intensity and number of cells stained positive as previously described^26^. Immunohistochemical analysis of normal breast showed complete absence of Axl staining, consistent with previous reports^25^, whereas nearly all ductal epithelium expressed moderate-high levels of Gas6. Axl protein slightly increased in DCIS (primarily tumor epithelial cells), with the most intense expression in IDC (Fig. 3a). Conversely, Gas6 protein expression trended downwards as lesions progressed to IDC (Figs 1c, 3), and there was a weak significant correlation between Gas6 and Axl protein scores (*r* = 0.27, Pearson’s correlation, *p* = 0.0004) (Fig. 3b). Although a subset of cells appeared to co-express Gas6 and Axl, the majority of the Gas6^+^ cells were Axl-negative (Fig. 3a). Next, we performed correlation tests using METABRIC and TCGA data sets. The correlation was significantly moderate between Gas6 mRNA and Axl mRNA when all patients were included without classification. We then performed the test on PAM50-classified groups; in METABRIC data set, the correlation between Gas6 mRNA and Axl mRNA was not significant in the non-cancerous group, yet it was significantly weak in normal-like patients, and moderate in Luminal A, Luminal B, Her2^+^, basal-like, and Claudin low patients. In TCGA data set, the correlation between Gas6 mRNA and Axl mRNA was not significant in normal-like patients, but it was significantly moderate in Luminal A, Luminal B, Her2^+^ and basal-like patients (Fig. 3c). These results suggest that epithelial Gas6 in normal breast and potentially breast cancer may have functions both dependent and independent of Axl.

### High Gas6 expression is associated with improved overall and RFS

**Fig. 4:**
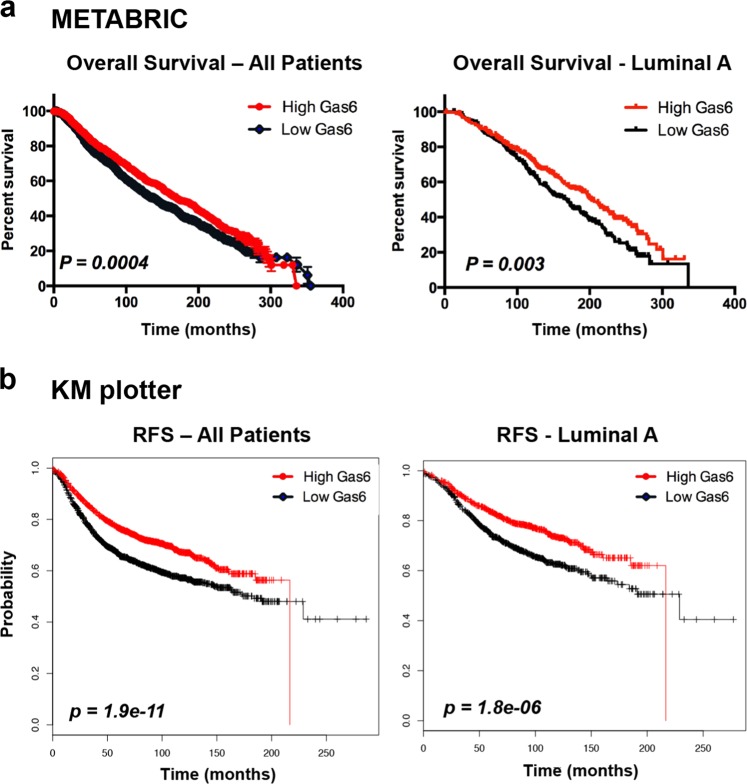
Higher Gas6 mRNA predicts better survival in breast cancer. **a** Kaplan–Meier plots generated from METABRIC show overall survival in all patients (*n* = 1904, *p* = 0.0004) (left) and in luminal A patients (*n* = 679, *p* = 0.003) (right), with high and low levels of Gas6 mRNA. **b** Kaplan–Meier plots from KMplotter representing relapse-free survival (RFS) of all patients (*n* = 3951, *p* = 1.9 × 10^−11^) (left), and in luminal A patients (*n* = 1933, *p* = 1.8 × 10^−6^) (right) with high and low levels of Gas6 mRNA. Log-rank, Chi-squared test for statistical analysis.

Given the higher levels of Gas6 in normal breast and DCIS, and reduction of Gas6 in invasive breast cancer, most prominently in several subtypes known to be aggressive, we asked whether Gas6 was associated with patient survival or prognosis. There was a significant improvement in overall survival in Gas6 high patients in the METABRIC data set in all patients/all treatments (*n* = 1904, *p* = 0.0004) (Fig. 4a). When stratified by subtype, there was a significant increase in overall survival in Gas6 high luminal A patients as compared with Gas6 low (*n* = 679, *p* = 0.003) (Fig. 4a), whereas all other subtypes showed no significant changes in overall survival (Supplementary Fig. 5a). In support, tumors with low Gas6 expression are larger in size, have higher cellularity, higher histological grade, and an increased Nottingham Prognostic Index (Table 2), all of which are prognostic markers for patient response to neoadjuvant chemotherapy^27^.

**Table 2:** Clinical attributes of patients with low and high levels of Gas6 in METABRIC data set.

Clinical attribute	Low Gas6 mRNA	High Gas6 mRNA	*p* value
Tumor Size (*n* = 1854*)*	26.99 ± 0.524	25.57 ± 0.466	0.043 (*)
Neoplasm histologic grade (*n* = 1780)	2.525 ± 0.022	2.32 ± 0.022	0.0001 (****)
Nottingham prognostic index (*n* = 1850)	4.195 ± 0.0367	3.891 ± 0.0377	0.0001 (****)
Cellularity (*n* = 1139)	9.663 ± 0.7805	9.150 ± 0.7762	0.0001 (****)

Next, we examined RFS in patients that received treatment, either chemical or hormonal, using the Kaplan–Meier plotter database^28^. RFS was superior in patients with tumors expressing higher levels of Gas6 mRNA, regardless of whether these patients received any treatment (*n* = 3951, *p* = 1.9 × 10^−11^) (Fig. 4b). Gas6 high patients with luminal A breast cancer showed significantly improved RFS over Gas6 low patients (*n* = 1933, *p* = 1.8 × 10^−6^) (Fig. 4b), whereas all other subtypes showed only moderate improvement to no change in RFS (Supplementary Fig. 5b). RFS was not significantly changed when patients that received treatment were stratified by grade (Supplementary Fig. 5b). Taken together, these results suggest that high Gas6 mRNA is associated with increased patient survival and is likely not predictive of poor outcome.

## Discussion

There has been enormous interest in targeting the TAMR family as a therapeutic strategy for metastatic breast cancer, and a number of tyrosine kinase inhibitors to Axl and Mer are currently in clinical trials^29^. Gas6 has primarily been shown to be a macrophage-derived factor that activates Axl in mouse models of breast cancer, and Warfarin, an anticoagulant that blocks the interaction between Gas6 and Axl, has recently been proposed as a cancer prevention strategy^30,31^. Despite the known tumor-promoting effects of macrophage Gas6 in vitro and in mouse models, little is known about the relevance and function of tumor-derived Gas6 in human breast cancer. The goals of this study were to identify Gas6 expression patterns in early and late stages of breast cancer and to determine whether tumor-derived Gas6 is associated with breast cancer progression.

We previously showed that Gas6 protein expression was high in mouse p53-null pre-invasive lesions but declined in established tumors^18,32^, suggesting that Gas6 has a major role in the transition from premalignancy to invasive cancer. Moreover, we showed that macrophage Gas6 induced a malignant phenotype in vitro by activating Axl on pre-invasive cells, however in vivo, once tumors were established, Gas6 was not required for further progression^18^. These results led us to directly address whether Gas6 protein expression decreased during human breast cancer progression. Here, we showed that Gas6 is highly expressed in the ductal epithelial cells of normal breast with faint stromal expression (Fig. 1), which is consistent with our previous studies of the normal murine mammary gland^19^. Epithelial-derived Gas6 protein expression was significantly increased in DCIS, but declined in invasive tumors (Fig. 1), supporting our published studies in mouse models^18^.

Early studies using a small cohort of patients showed that Gas6 mRNA is expressed in human breast cancer and correlates with progesterone receptor and favorable clinical parameters^33^. In support of these findings, we found that Gas6 showed the lowest protein expression in TNBC when stratified by subtype (Fig. 2a). Analysis of the METABRIC and TCGA data sets showed the highest Gas6 mRNA levels in luminal A breast cancer and then a decline towards luminal B, Her2^+^, and basal-like (Fig. 2b, c). luminal A breast cancer typically has the best prognosis among other subtypes, considering the response to therapy and the slow growth of cancer cells attributed to the low levels of Ki67 expression. Luminal B and Her2^+^ subtypes generally have lower overall survival owing to their fast growth and tendency to relapse after removal. Likewise, basal-like breast cancer is associated with poor overall survival, partly due to increased metastatic potential and a lack of targeted therapeutic strategies^22,34,35^, although a proportion of these patients are associated with good prognosis^36^. These observations led us to ask whether Gas6 expression in human breast cancers correlates with overall survival. Analysis of the METABRIC data set that includes all patients regardless of treatment revealed a significant increase in overall survival in patients with high Gas6 mRNA (Fig. 4a). Using the Kaplan–Meier plotter database (KMplotter)^28^, we found that high Gas6 mRNA correlated to improved RFS in breast cancer patients, particularly in patients with luminal A breast cancer, when stratified with those who had received therapy (Fig. 4b). These findings suggest that Gas6 protein or mRNA expression in human breast tumors may not be a negative prognostic factor.

Similar to earlier studies associating Gas6 expression with favorable clinical outcome in breast cancer, Gas6 was shown to inhibit intestinal tumorigenesis in a mouse model of intestinal cancer, and Gas6 protein expression in human colorectal cancers positively correlated with prognosis^37^. However, numerous other reports suggest that Gas6 is tumor promoting in various cancers including colon, thyroid, lung, and ovarian (reviewed in ref. ^38^). In breast cancer, studies have largely focused on Axl, and targeting Axl or Gas6-Axl has been proposed. Axl has been correlated with poor prognosis in breast cancer patients and staining of clinical samples showed that Axl is overexpressed in a small percentage of TNBC and Her2^+^ patients^13,25^. In this study, tumor-derived Gas6 protein did not strongly correlate to Axl expression (Fig. 3). In support, numerous studies have shown Gas6-independent activation of Axl in cancer. Goyette et al. showed that genetic ablation of Axl in MMTV-Neu tumors results in decreased tumor formation and metastasis, although Gas6 deletion had no effect on mammary tumorigenesis^25^. Protein S, which is highly homologous to Gas6, was shown to regulate Axl and drive tumorigenesis in a mouse model of oral squamous cell carcinoma^39^. Protein S was also shown to activate Axl in glioblastoma, contributing to growth of aggressive tumors^40^. Additional Gas6-independent mechanisms involving interactions of Axl with other RTKs including EGFR^41^, MET^42^, and VEGFR2^43^ have also been described. Thus, it is plausible that Axl-mediated breast cancer progression may not require Gas6. It is also possible that low levels of Gas6 may be required to sustain Axl activation in tumor cells. Additional functional studies are required to fully understand these mechanisms.

In addition to reports of ligand-independent Axl-induced tumor progression, numerous studies suggest that targeting Gas6 or Gas6/Axl in breast cancer is beneficial. In ovarian cancer cell lines derived from mesenchymal (Mes) or epithelial (Epi-A) mouse models, Gas6-stimulated Axl-RTK crosstalk in Mes cells, whereas Gas6-induced Axl activation did not involve other RTKs in Epi-A cells^44^. Leukocyte-derived Gas6 was shown to mediate tumor growth in a syngeneic TNBC mouse model (4T1) as well as in other cancer cell models^12^. Kariolis et al.^45^ described an Axl “decoy receptor” that sequesters Gas6 and was shown to inhibit metastasis in ovarian cancer xenografts and a syngeneic TNBC mouse model (4T1). Of note, the decoy receptor not only blocks the Gas6-Axl interaction, but also other Gas6 receptors, including Mer. In another study, dual inhibition of Gas6 and Mer decreased tumor-associated macrophages, increased CD4^+^ T cells and reduced tumor formation in lung cancer cells in vivo^46^. Gas6 has also been shown to enhance T regulatory cell (Treg) suppressor activity by binding Axl on Tregs^47^. In the present study, our data showed that stromal Gas6 expression did not significantly change during breast cancer progression (Supplemental Fig. 1), however it remains unclear as to whether macrophage-derived Gas6, or Gas6 from other stromal cells, has tumor-promoting activities. Despite the observed decreased in tumor-derived Gas6 in advanced breast cancers, targeting stromal Gas6 and its receptors remains a promising therapeutic approach and may be valuable when combined with immunotherapies^40,48^. Further studies are required to understand the biological function of Gas6 in human breast cancer.

## Methods

### Patient samples and ethics

A TMA (BC081116C) containing samples of 100 patient tissues of various breast cancer subtypes and nine adjacent normal breast (referred to as “normal” throughout the manuscript) were purchased from US Biomax (Biomax website states that tissues were collected upon the donors written consent under HIPPA approved protocols). TMAs containing 12 patient samples with DCIS, and 44 IDC patient samples with the corresponding IDC-associated DCIS, were kindly obtained from Dr. Fariba Behbod (University of Kansas Medical Center, KS, USA). All patients gave written informed consent for participation in this University of Kansas Medical Center Institutional Review Board–approved study allowing collection of additional biopsy and or surgical tissue for research. Histopathological characteristics are listed in Table 1.

### Immunostaining

Paraffin-embedded TMAs sections (5 μm) and paraffin-embedded cell pellets (NCI-H226, HT29, H1299 and Jurkat cells (Cell signaling) were deparaffinized, rehydrated, and antigen retrieval was performed in the microwave using 10 mM sodium citrate for 20 mins. For immunohistochemistry (IHC), endogenous peroxidases were quenched with 3% H_2_O_2_ in methanol, blocked with 5% bovine serum albumin (BSA) in PBS containing 0.05% Tween and incubated with a Gas6 antibody (R&D #AF885, 1:50 dilution) and Axl antibody (Cell Signaling Technology #8661 S, 1:3000 dilution) overnight at 4 °C. The next day, slides were washed with PBS and incubated with a biotinylated antibody (1:500) (Vector Laboratories) for 30 min. Slides were washed with PBS, incubated for 10 min with the VECTASTAIN Elite ABC-HRP reagent, R.T.U (Vector Laboratories), and developed using a DAB peroxidase substrate kit (Vector Laboratories). Sections were then counterstained with hematoxylin, dehydrated and mounted with permount (Fisher Scientific). Images were acquired using a Nikon Eclipse microscope (Nikon Instruments). For immunofluorescence, sections were blocked with 5% BSA in PBS containing 0.05% Tween for 1 h at RT and incubated overnight at 4 °C, with Gas6 antibody (1:50 dilution) and Axl antibody (1:3000 dilution). The next day, slides were washed with PBST, and incubated in dark with fluorescent 2ry antibody (1:500, Alexaflour 488, Thermo Scientific) for 1 h. Slides were then washed with PBST and nuclei were counterstained (Vector laboratories). Fluorescent images were captured with Nikon confocal microscope (Nikon instruments). For IHC antibody scoring, tissues were examined using ×20 and ×40 objectives. An arbitrary number was given on a scale of 0–3 by visual examination, based on the intensity and the number of stained cells, taking any field to field variation into account, where 0 was assigned for negative staining, 1 for low, 2 for moderate, and 3 for strong staining^26^. A separate score was assigned for either epithelial or stromal staining, and all samples were scored blindly by two independent individuals.

### Online data and statistical analyses

Epithelial Gas6 score in TMAs sections was statistically analyzed using Chi-squared test for pair-wise comparison. Data were considered statistically significant if *P* values ≤ 0.01. A Pearson’s correlation coefficient was calculated to assess the relationship between the scores of Gas6 and Axl protein expression using GraphPad Prism8. Gas6 mRNA expression in normal and tumor tissues was gathered from GEPIA, an online platform with RNA-seq data from TCGA and GTEx databases^20^. Gas6 transcripts per million from both normal and tumor tissue were plotted using one-way analysis of variance (ANOVA) differential method and a *q* value cutoff of 0.01. METABRIC and TCGA data were accessed through cBioportal and was further categorized using the Pam50 classification^22^. Patient Gas6 mRNA levels were matched with the appropriate sample-ID. With median Gas6 expression as the cutoff value, GraphPad Prism software was used to calculate statistical differences of mean Gas6 expression between normal and breast cancer subtypes using one-way ANOVA with post hoc Dunnett’s multiple comparison test. Correlating Gas6 mRNA and Axl mRNA in METABRIC and TCGA datasets was performed using the Pearson’s correlation module in GraphPad Prism, and using Gas6 and Axl mRNA values from PAM50-classified patients subtypes. Survival curves were generated using two data sets: METABRIC data set was mined and the overall survival status of patients with different subtypes and the corresponding Gas6 mRNA level per patient were downloaded and grouped as high and low, based on Gas6 mRNA expression level and using the median expression as a cutoff. Survival graphs were then plotted using survival module in GraphPad Prism8. The second dataset was Kaplan–Meier Plotter (KMplotter), an online platform combining gene microarray data and patient survival rates from Gene Expression Omnibus (Affymetrix HGU133A and HGU133 + 2 microarrays)^28^. Patients were divided using an auto selection feature based on median and quartile expression levels of Gas6 (valid Affy ID: 1598_g_at) and quality controlled for redundant samples and biased assays. Median survival was reported in months and compared for significance with a hazard ratio and *p* value generated on the graph. A *p* value of <0.05 was considered statistically significant (Log-rank, Chi-squared test). Overall survival and RFS were tested without further criteria filtering. RFS for subtypes were restricted to treated patients cohort, and the subtypes selection was an intrinsic grouping of patients based on their gene expression.

### Reporting summary

Further information on research design is available in the Nature Research Reporting Summary linked to this article.

## Supplementary information

supplementary materials

reporting summary

## Data Availability

Clinical and histopathological data for the TMAs acquired from the US Biomax can be accessed from the US biomax website (https://www.biomax.us/tissue-arrays/Breast/BC081116c) (Table 1). GEPIA data set analyzed in this study can be accessed via the website (http://gepia.cancer-pku.cn/index.html) (Supplementary Fig. 2). The METABRIC and the TCGA breast cancer data sets analyzed during this study can be accessed from the cBioPortal for Cancer Genomics repository (https://www.cbioportal.org/) (Figs 2b, c, 4a, and Table 2). KM plotter data set analyzed in this study can be accessed from KM plotter website (http://kmplot.com/analysis/index.php?p=service) (Fig. 4b and Supplementary Fig. 5).
