# Supplementary material for: Gas6 expression is reduced in advanced breast cancers
Source: NPJ Precis Oncol. 2020 Apr 24;4:9. doi: 10.1038/s41698-020-0116-z (PMC7181799; doi:10.1038/s41698-020-0116-z)
Supplement: Supplementary file 1 — supplementary materials [file 41698_2020_116_MOESM1_ESM.pdf]

Supplementary Figure 1

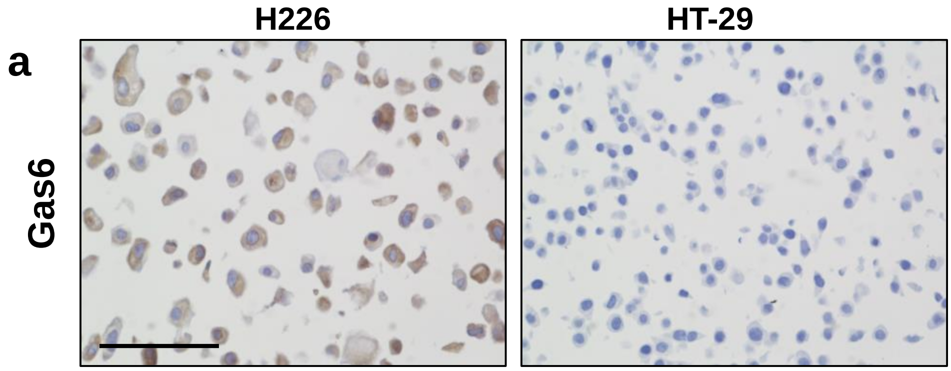

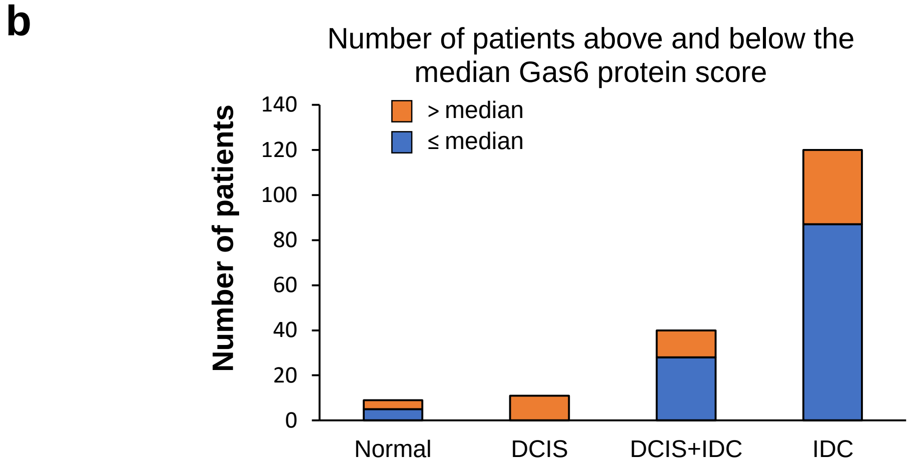

Chi-squared test comparing normal adjacent vs all other groups.

Normal versus	Unadjusted p from exact c^2	Adjusted p-value
DCIS	0.0081	significant
DCIS+IDC	0.5459	ns
IDC	0.2448	ns

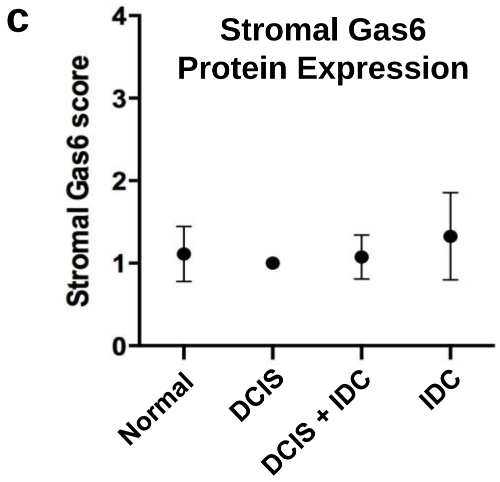

Supplementary Figure 1: Gas6 protein expression during breast cancer progression
a Immunohistochemical staining of Gas6 in paraffin-embedded cell pellets: NCI-H226 (Gas6 positive control) and HT-29 (negative control). Scale bar=50 μm. **b** Graph shows the number of patients above and below the median Gas6 score. Table shows the corresponding pairwise comparison using Chi-square analysis to assess statistical significance between normal and each of the other groups. ns = nonsignificant. **c** Representation of stromal Gas6 score (0: negative, 1: low, 2: moderate and 3: high) in normal, pure DCIS, DCIS + IDC, and IDC alone. Error bars are SEM, and Chi-squared analysis showed no statistical difference between normal breast and other tissues.

Supplementary Figure 2

a

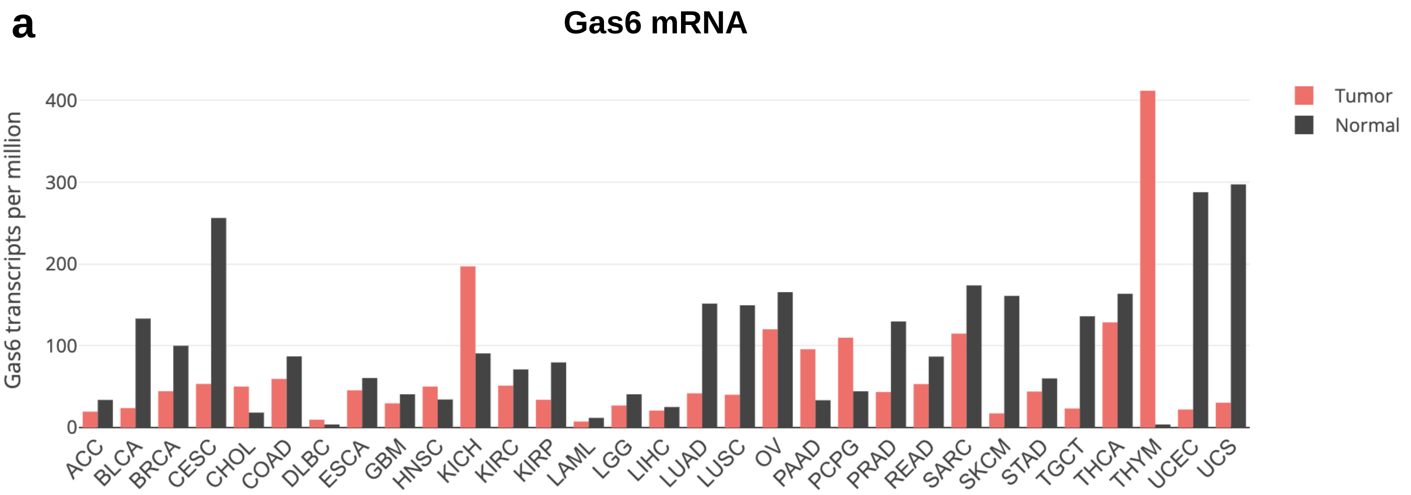

ACC Adrenocortical carcinoma
BLCA Bladder Urothelial Carcinoma
BRCA Breast invasive carcinoma
CESC Cervical squamous cell carcinoma and endocervical adenocarcinoma
CHOL Cholangio carcinoma
COAD Colon adenocarcinoma
DLBC Lymphoid Neoplasm Diffuse Large B-cell Lymphoma
ESCA Esophageal carcinoma
GBM Glioblastoma multiforme
HNSC Head and Neck squamous cell carcinoma
KICH Kidney Chromophobe
KIRC Kidney renal clear cell carcinoma
KIRP Kidney renal papillary cell carcinoma
LAML Acute Myeloid Leukemia
LGG Brain Lower Grade Glioma
LIHC Liver hepatocellular carcinoma
LUAD Lung adenocarcinoma
LUSC Lung squamous cell carcinoma
OV Ovarian serous cystadenocarcinoma
PAAD Pancreatic adenocarcinoma
PCPG Pheochromocytoma and Paraganglioma
PRAD Prostate adenocarcinoma
READ Rectum adenocarcinoma
SARC Sarcoma
SKCM Skin Cutaneous Melanoma
STAD Stomach adenocarcinoma
TGCT Testicular Germ Cell Tumors
THCA Thyroid carcinoma
THYM Thymoma
UCEC Uterine Corpus Endometrial Carcinoma
UCS Uterine Carcinosarcoma

b

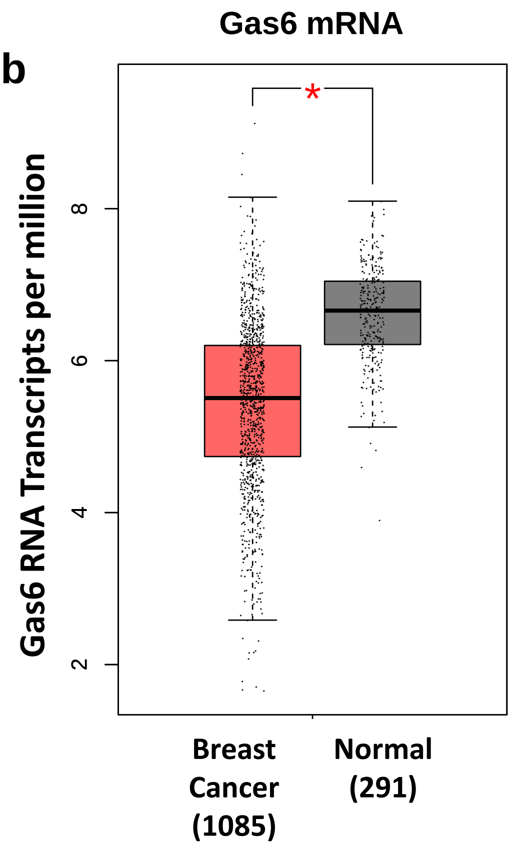

Supplementary Figure 2: Gas6 mRNA in normal and cancer tissues

a Gene expression profile across all tumor samples and paired normal tissues. Bars represent the median expression of tumor type or normal tissue. **b** A boxplot showing the mean transcripts per million for Gas6 mRNA in 291 normal breast tissues in contrast to 1085 from breast cancer tissues showing a significant reduction in Gas6 RNA in cancer tissues (unpaired t-test, *p<0.01). Graphs were generated from GEPIA database.

Supplementary Figure 3

a

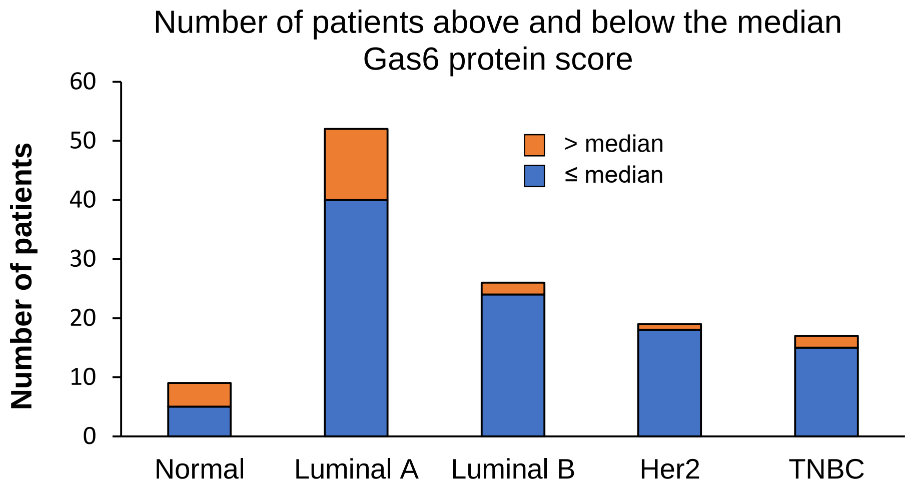

Chi-squared test comparing Normal adjacent vs all other groups.

Normal versus	Unadjusted p from exact c^2	Adjusted p-value
Luminal A	0.2964	ns
Luminal B	0.0123	ns
Her2	0.0254	ns
TNBC	0.0879	ns

b

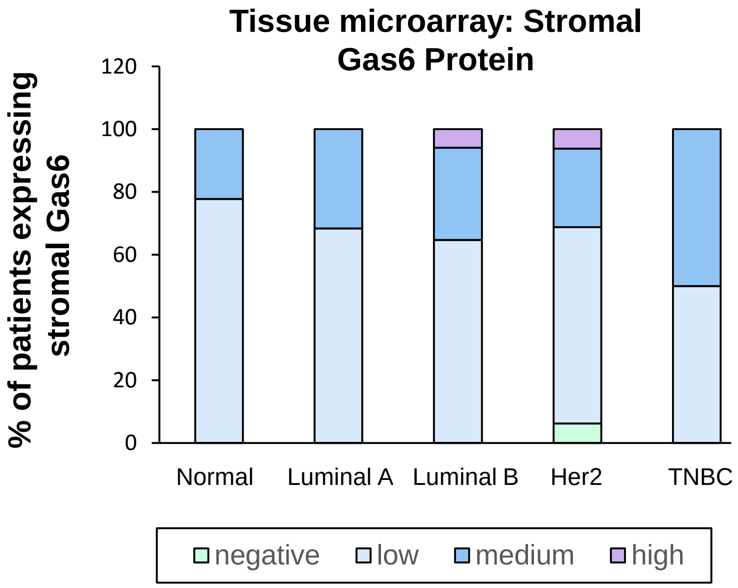

Supplementary Figure 3: Gas6 protein expression in breast cancer subtypes
a Number of patients above and below the median Gas6 score, and the corresponding pairwise comparison using Chi-squared test (table) to assess statistical significance between normal breast and each of the other groups. ns = not significant. **b** Graph depicts quantitation Gas6 protein staining of TMAs, expressed as the percentage (%) of patients expressing epithelial Gas6 protein in different molecular subtypes: normal breast (n=10), luminal A (n=39), luminal B (n=18), Her2^{+} (n= 19), and TNBC (n=19).

Supplementary Figure 4

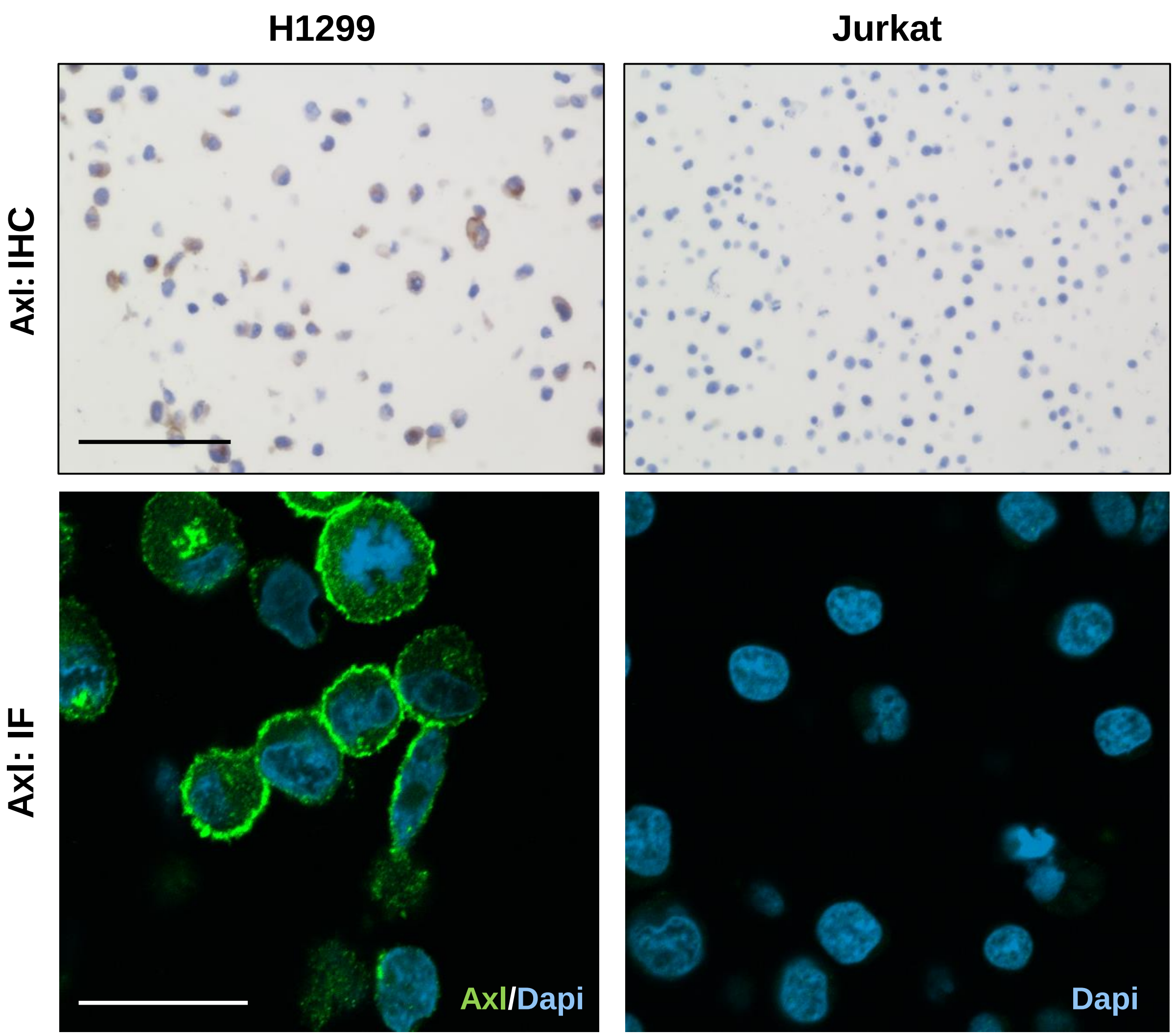

Supplementary Figure 4: Axl antibody validation in paraffin-embedded cell pellets
Images show immunohistochemical (top) and immunofluorescent (bottom) staining using the same antibody to Axl in paraffin-embedded cell pellets H1299 (Axl-positive cell line) and Jurkat (Axl-negative cell line). Scale bar=50 μm (top), 20 μm (bottom).

Supplementary Figure 5

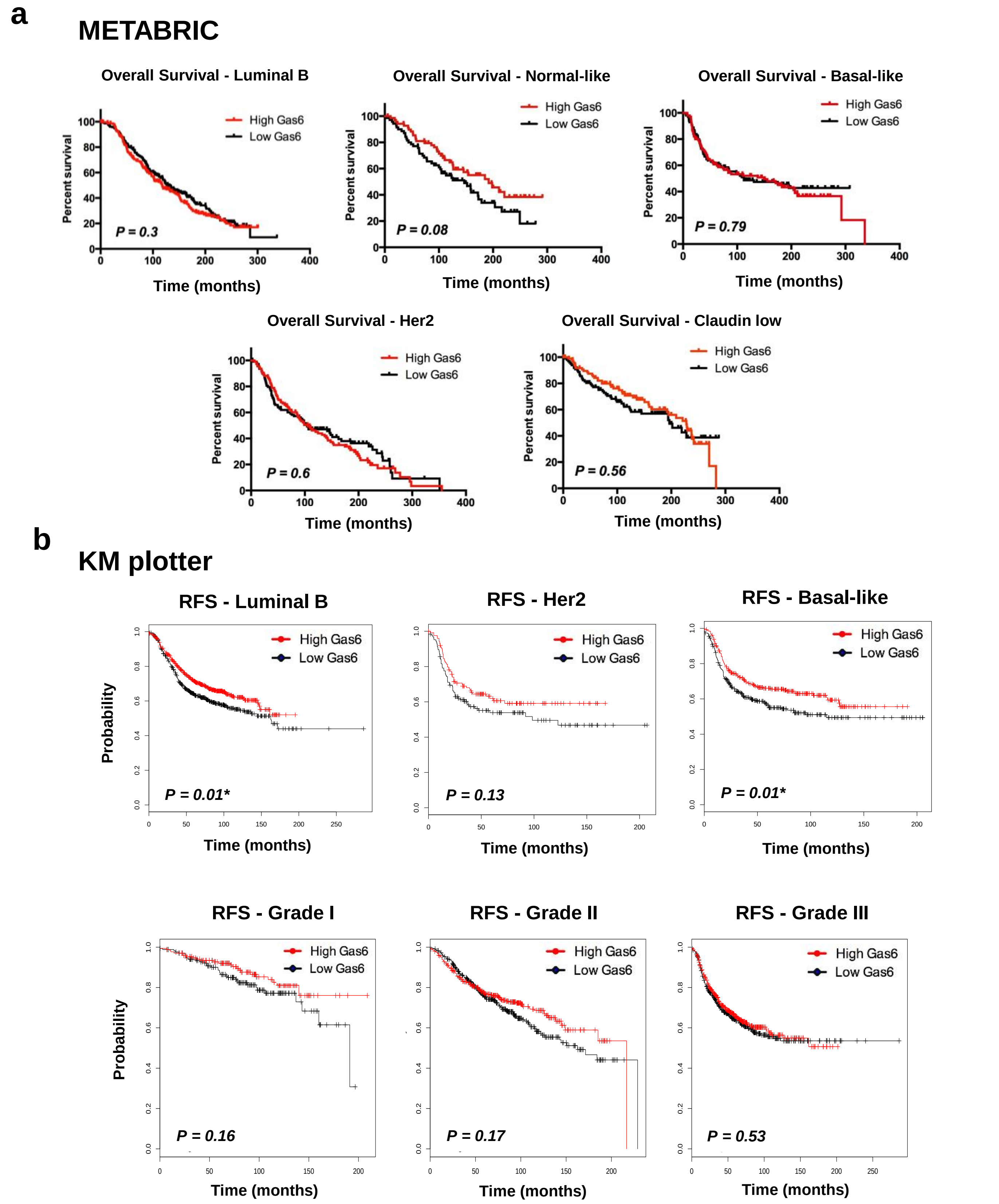

Supplementary Figure 5: Survival status of patients classified by subtype expressing high or low Gas6 mRNA. a Overall survival in luminal B, normal-like, Her2^{+}, basal-like and claudin low patients, with high and low levels of Gas6 mRNA. Graphs were generated using METABRIC dataset. **b** Kaplan Meier plots representing RFS of treated breast cancer luminal B, Her2^{+} and basal-like patients, in addition to RFS of treated patients with Grades I, II and III. Log-rank, Chi squared test for statistical analysis.
